# Kaiso, a transcriptional repressor, promotes cell migration and invasion of prostate cancer cells through regulation of miR-31 expression

**DOI:** 10.18632/oncotarget.6801

**Published:** 2015-12-30

**Authors:** Honghe Wang, Wei Liu, ShaNekkia Black, Omari Turner, Juliet M. Daniel, Windy Dean-Colomb, Qinghua P. He, Melissa Davis, Clayton Yates

**Affiliations:** ^1^ Department of Biology and Center for Cancer Research, Tuskegee University, Tuskegee, AL, USA; ^2^ Laboratory of Comparative Carcinogenesis, Cancer Center of Research, Frederick, MD, USA; ^3^ Department of Chemical Engineering, Tuskegee University, Tuskegee, AL, USA; ^4^ Department of Genetics, University of Georgia, Athens, GA, USA; ^5^ Department of Biology, McMaster University, Hamilton, ON, Canada; ^6^ Department of Oncologic Research, University Hospital and Clinics, Lafayette General Health, Lafayette, LA, USA

**Keywords:** miRNA, Kaiso, DNA methylation, prostate cancer

## Abstract

Kaiso, a member of the BTB/POZ zinc finger protein family, functions as a transcriptional repressor by binding to sequence-specific Kaiso binding sites or to methyl-CpG dinucleotides. Previously, we demonstrated that Kaiso overexpression and nuclear localization correlated with the progression of prostate cancer (PCa). Therefore, our objective was to explore the molecular mechanisms underlying Kaiso-mediated PCa progression. Comparative analysis of miRNA arrays revealed that 13 miRNAs were significantly altered (> 1.5 fold, *p* < 0.05) in sh-Kaiso PC-3 compared to sh-Scr control cells. Real-time PCR validated that three miRNAs (9, 31, 636) were increased in sh-Kaiso cells similar to cells treated with 5-aza-2′-deoxycytidine. miR-31 expression negatively correlated with Kaiso expression and with methylation of the miR-31 promoter in a panel of PCa cell lines. ChIP assays revealed that Kaiso binds directly to the miR-31 promoter in a methylation-dependent manner. Over-expression of miR-31 decreased cell proliferation, migration and invasiveness of PC-3 cells, whereas cells transfected with anti-miR-31 restored proliferation, migration and invasiveness of sh-Kaiso PC-3 cells. In PCa patients, Kaiso high/miR-31 low expression correlated with worse overall survival relative to each marker individually. In conclusion, these results demonstrate that Kaiso promotes cell migration and invasiveness through regulation of miR-31 expression.

## INTRODUCTION

For men, PCa is the most frequently diagnosed cancer and a leading cause of cancer death, with the mortality and morbidity being mainly due to tumor invasion and metastasis [1]. Current therapies are often effective against localized PCa; however, once the tumor invades and disseminates to surrounding tissues or metastasizes to distant sites, available treatments prolong patient survival only slightly [2]. Thus, it is necessary to unravel the genetic and epigenetic molecular regulation associated with this transition and to develop a rational approach to treatment.

Epigenetic silencing of tumor suppressor genes contributes to the pathogenesis of various cancers, including PCas. DNA methylation, a common epigenetic change, results from changes in cytosine methylation, typically at cytosine-guanine dinucleotides (CpG), or from changes in DNA-associated proteins. Like their gene counterparts, microRNAs (miRNAs) are also subject to epigenetic regulatory mechanisms such as promoter CpG island hypermethylation and transcriptional deregulation or repression [3, 4]. Promoter hypermethylation has been demonstrated for numerous miRNAs, including the miR-200 family, miR-34a, and miR-31, which are responsible for various cancer-related events, such as evading apoptosis, increased cell proliferation, and the epithelial-mesenchymal transition [5–8]. To date most studies have focused on the genes targeted by miRNA, and thus the molecular mechanisms that regulate expression of miRNAs are not yet well defined.

The expression and localization of Kaiso, a transcriptional repressor that belongs to the BTB/POZ zinc-finger protein family, correlate with several human malignancies, including breast, prostate, lung, and colorectal cancers [9–12]. Like many other POZ proteins, Kaiso has the characteristic POZ domain at its amino-terminus, where it facilitates Kaiso homodimerization and heterodimerization with various proteins; the zinc finger domain at the carboxy-terminus of Kaiso is responsible for DNA association [12] Unlike the previously characterized POZ proteins, however, Kaiso recognizes sequence-specific Kaiso binding sites or methyl-CpG dinucleotides [13–15]. Recently, we reported that, in high-grade and metastatic prostate and breast cancers, Kaiso expression shifts from the cytoplasm to the nucleus [16]. At the cellular level, this shift in localization causes methylation-dependent silencing of E-cadherin, which in turn promotes increased cell migration and invasiveness; these are characteristics of highly aggressive tumors [16]. In both breast and colon cancer cell lines, Kaiso is responsible for silencing the cell cycle-related genes, cyclin D1 and CDKN2A [10, 17]. Since Kaiso is involved in methylation-dependent silencing of various tumor-related genes, we investigated Kaiso's function in the regulation of expression of hypermethylated miRNA.

In this report, we describe several miRNAs that are silenced through DNA methylation and are also regulated by Kaiso. Using a genome-wide miRNA array, which was validated by qPCR analysis and ChIP assays, miR-31 was identified as a target of Kaiso in PCa in a methylation-dependent manner. Moreover, in a panel of PCa cell lines, miR-31 expression negatively correlated with Kaiso levels. Re-expression of miR-31 in the prostate carcinoma PC-3 cells inhibited cell proliferation, migration and invasiveness. Furthermore, Kaiso high/miR-31 low expression correlated with worse overall survival in PCa patients, relative to miR-31 or Kaiso expression alone. Thus, these results indicate that Kaiso regulates the expression of hypermethylated miR-31 in PCa. These results further highlight the oncogenic role of Kaiso in PCa.

## RESULTS

### Identification of miRNAs epigenetically regulated by Kaiso in PC-3 cells

**Figure 1:**
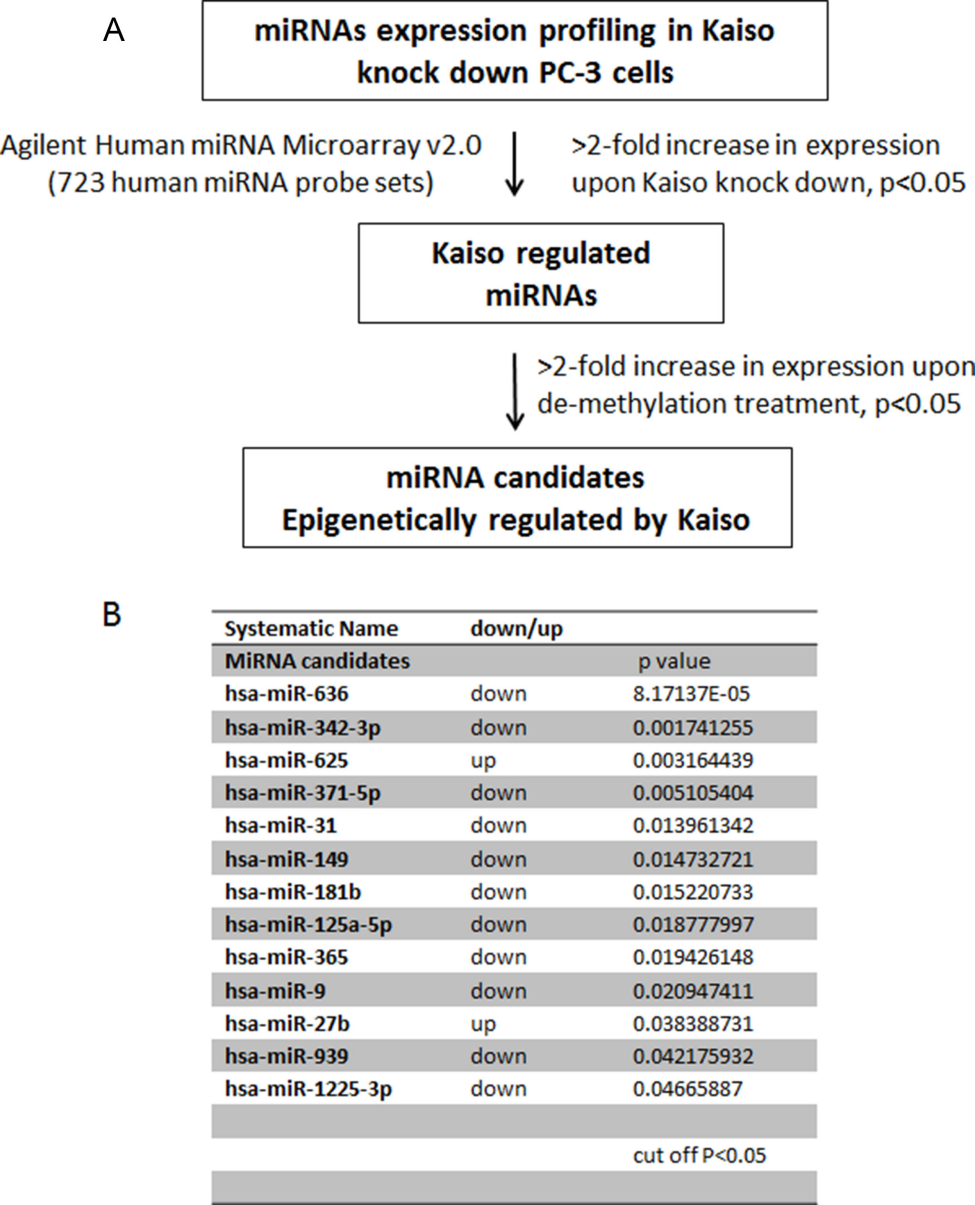

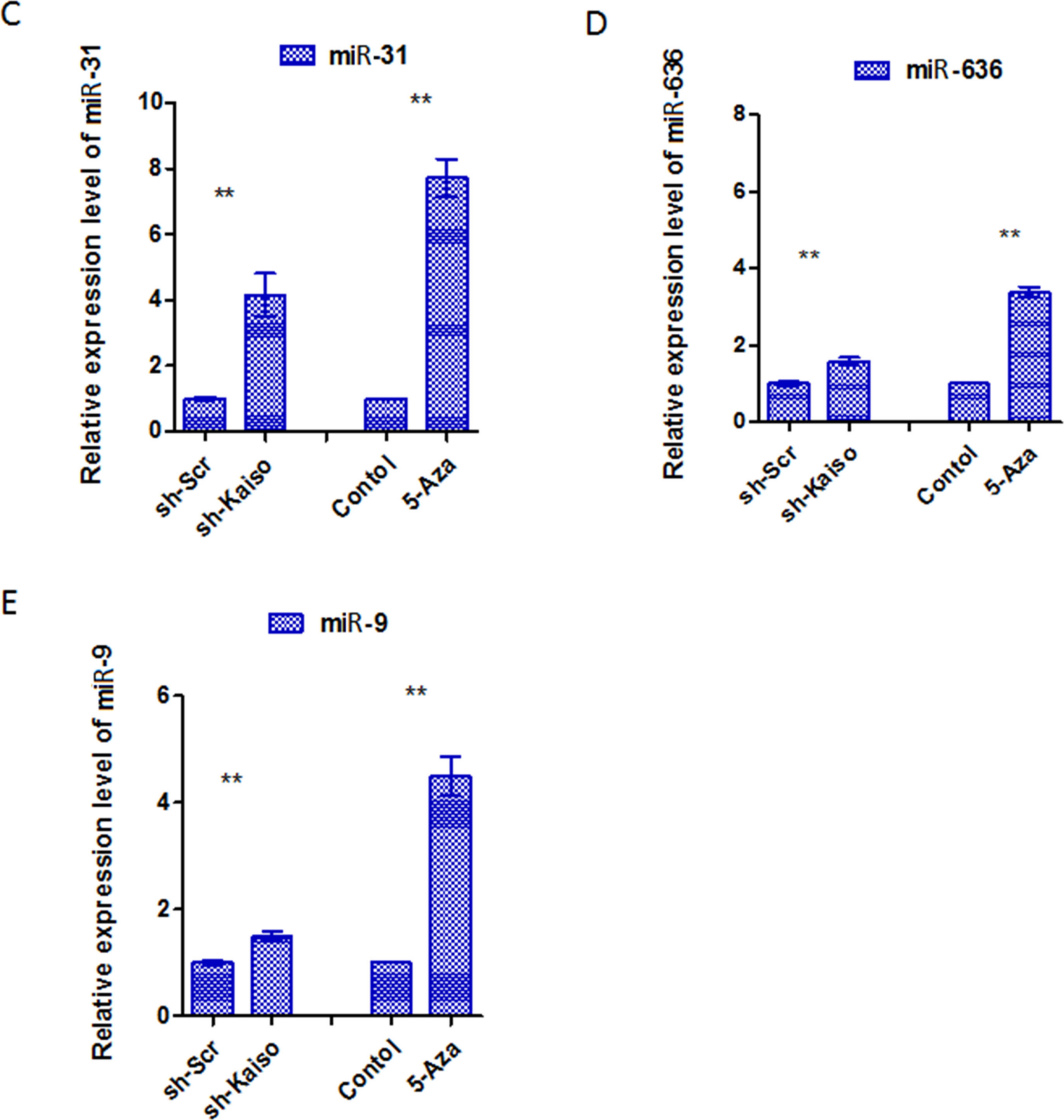
The screening and validation of miRNAs epigenetically regulated by Kaiso (**A**) Schematic presentation of the screening for miRNAs altered in PC-3 sh-Scr cells vs sh-Kaiso cells. (**B**) miRNAs altered in sh-Kaiso cells compared to PC-3 sh-Scr cells, *p* < 0.05 (Kaiso expression in sh-Kaiso PC-3 was determined by immunoblot (Supplementary Figure 1A). (**C–E**) Expression of miR-9, miR-636, and miR-31 in PC-3 cells untreated or exposed to the 500 nM 5-aza-2′-deoxycytidine (5-aza) and sh-Kaiso and sh-Scr (control) PC-3 cells analyzed by qRT-PCR. Data were normalized to a U6 snRNA control. *p < 0.05, **p < 0.01.

Previously, we demonstrated that Kaiso is responsible for methylation-dependent transcriptional silencing of various genes in both PC-3 and DU-145 cells that were stably transfected with sh-Kaiso pRFP-C-RS plasmid or control plasmid (sh-Scr) [16] (Supplementary Figure 1). Since Kaiso is a transcriptional repressor, we proposed that it also regulates the expression of miRNAs, which in turn, repress expression of target genes. To determine if Kaiso regulates expression of miRNAs in PCa, the expression of miRNA transcripts in sh-Kaiso PC-3 cells were compared to those of scr-PC-3 control cells by use of Agilent miRNA array analysis. Differentially expressed miRNAs were chosen based on at least 1.5-fold changes and a non-stringent *p* cutoff (*p* < 0.05) (Figure 1A). Overall, 13 miRNAs differed in expression, of which 11 microRNAs were up-regulated, and 2 microRNAs were down-regulated (Figure 1B). To identify miRNA candidates that are epigenetically silenced by Kaiso, PC-3 cells were treated with 500 nM 5-aza-2′-deoxycytidine (5-aza) for 96 hr and qRT-PCR was performed to validate epigenetically silenced miRNAs found in the miRNA microarray. Of the 11 miRNAs that were validated by qRT-PCR. miR-31, miR-636, and miR-9 displayed significantly differential expression (Figure 1C, 1D, 1E) both in PC-3 cells treated with 5-aza or Kaiso knockdown; the others showed less or no significant changes (data not shown).

### miR-31 expression is inversely correlated with Kaiso expression

**Figure 2:**
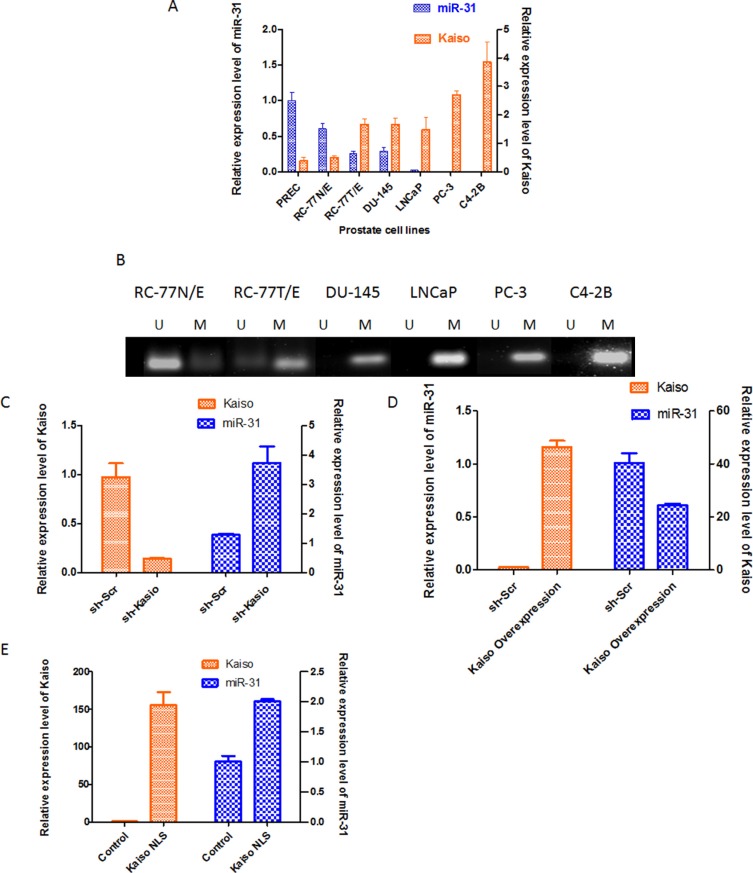
miR-31 expression is reversely correlated with kaiso expression in prostate cancer cells (**A**) Correlation of Kaiso and miR-31 levels in a panel of PCa cell lines. Levels of Kaiso mRNA where determined by qRT-PCR, with hypoxanthine-guanine phosphoribosyltransferase as the loading control. miR-31 expression levels were determined by qRT-PCR and normalized to the U6 snRNA control; *n* = 4 ± S.E (*p < 0.05). (**B**) Composite gel of MSP of the miR-31 promoter demonstrates that malignant cell lines have miR-31 promoter hypermethylation (M) relative to non-malignant cells, which have an unmethylated (U) miR-31 promoter. (**C**) DU-145 cells were transfected with sh-Kaiso or the vector control (sh-Scr). Kaiso expression was determined by qRT-PCR and immunoblots (Supplementary Figure 1B). (**D**) Cells with over-expressed Kaiso have decreased expression of miR-31. (**E**) DU-145 cells transfected with the Kaiso-NLS plasmid showed increased miR-31 expression compared to control vector. Data are expressed as means ± S.E. of three independent experiments.

**Figure 3:**
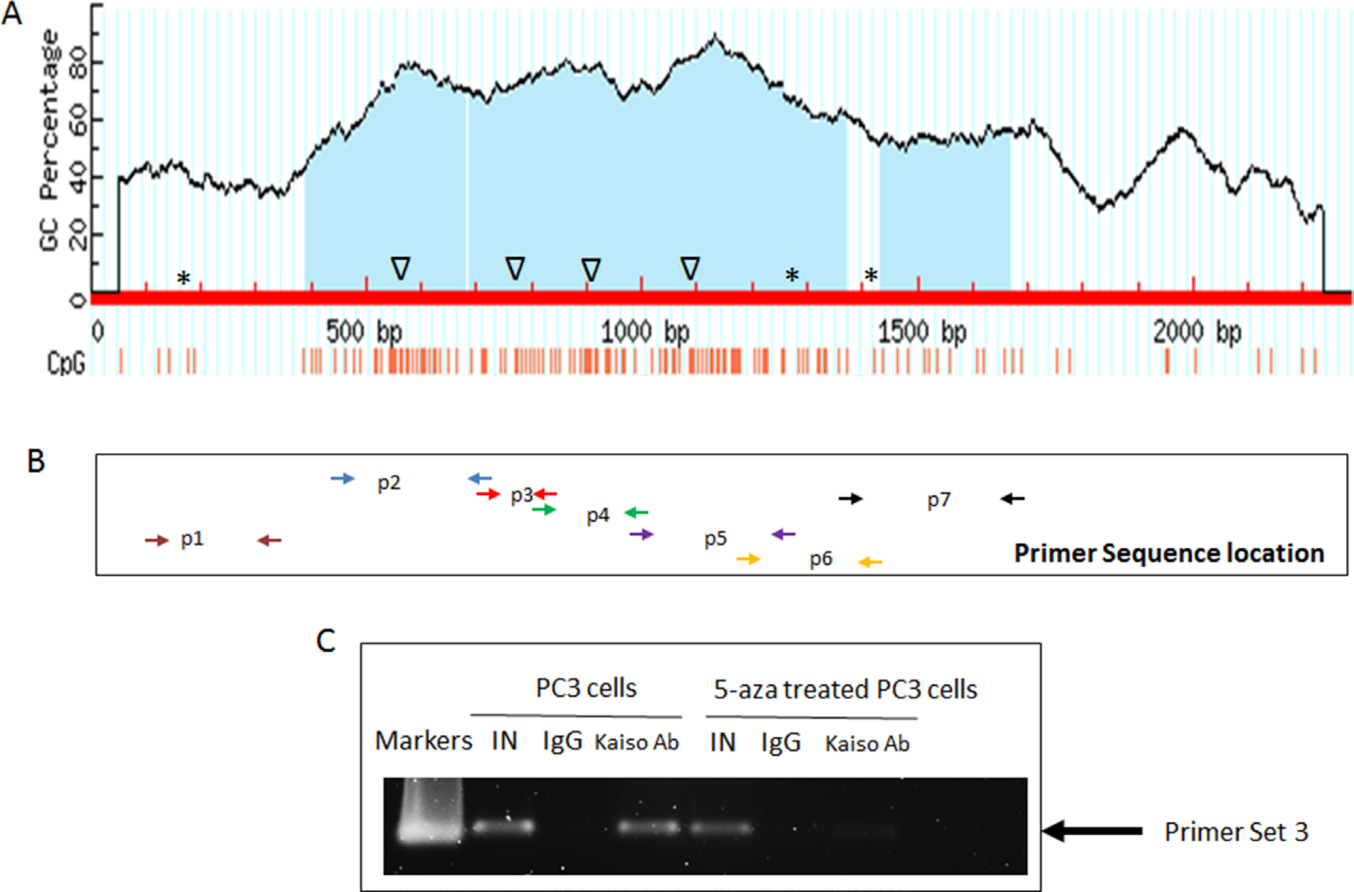
Kaiso binds to CpG islands in the *miR-31* promoter regions (**A**) Schematic map of the CpG islands and potential Kaiso MSB/CBS binding sites in the *miR-31* promoter. Δ: MSB; *CKBS. (**B**) Seven pairs of primers that cover the CpG islands in miR-31 promoter region. (**C**) ChIP analysis of the association between Kaiso protein and the miR-31 promoter sequence, in the presence or absence of 500 nM 5-aza-2′-deoxycytidine (5-aza). To control for specificity of the antibody used, mouse IgG was used as a negative control. Anti-RNA Polymerase antibody served as a positive control; The graph represents results from PCR amplification of the *miR-31* promoter amplified from the anti-Kaiso or anti-RNA Polymerase immunoprecipitates. One-tenth of the input sample was used for PCR amplification. IN, input; IP, immunoprecipitation; Kaiso Ab, immunoprecipitation with Kaiso Ab; PC: Positive control with anti-RNA Polymerase antibody; NDC, No DNA control.

miR-31 is down-regulated in breast cancers, lung cancers, and PCa [22–24], suggesting that it functions in tumor progression. Since Kaiso over-expression correlates with tumor progression and metastasis [16, 21], we choose to study miR-31 further by determining the correlation between Kaiso and miR-31 in a panel of human prostate cell lines (normal cell line, PREC; immortal normal epithelial cell line, RC-77N/E; and PCa cell lines RC77T/E, DU-145, PC-3, LNCaP, and C4–2B). Endogenous expression of miR-31 in PREC cells and RC-77N/E cells was higher than in the PCa cell lines, with a decreasing trend in the more aggressive cell lines (Figure 3A). Kaiso mRNA expression was low in PREC and RC-77N/E cells and high in PCa cell lines, with increased expression in the more aggressive cell lines like PC-3 and C4–2B cells (Figure 2A Upper Panel). To determine if the decreased miR-31 in PCa cell lines is due to hypermethylation of the miR-31 promoter, MSP for miR-31 was performed for representative cell lines with multiple primers (Supplementary Table 1). Non-malignant RC-77N cells had an unmethylated promoter, but the malignant RC-77T/E cells, LNCaP cells, and the more aggressive DU-145, PC-3, and C42B cells had methylated promoters (Figure 2A Lower Panel). To further determine the miR-31/Kaiso relationship, we performed real-time PCR on validated miR-31 target genes, ITGA5, MMP16, RDX, Fzd3, CXCL12, and SRC in our sh-Kaiso model. Interestingly, MMP16, Fzd3, and Src showed significant decreases in expression compared to sh-Scr cells (Supplementary Figure 2).

We previously found in PCa patients that cytoplasmic-to-nuclear shuttling of Kaiso is associated with more aggressive tumors [16]. To determine if subcellular localization has an effect on miR-31 expression, DU-145 cells, which express low levels of miR-31 and have Kaiso predominately in the cytoplasm [7], were utilized [16]. DU-145 cells transfected with sh-Kaiso had reduced expression of Kaiso but elevated levels of miR-31 (Figure 2B). Those transfected with the full-length Kaiso plasmid (Supplementary Figure 3) demonstrated an increase in Kaiso expression and a decrease in miR-31 expression (Figure 2C). DU-145 cells transfected with a Kaiso-NLS plasmid (with mutant Nuclear Localization Sequence [25] similarly demonstrated an increase in Kaiso expression. These cells also showed increased miR-31 expression relative to control cells (Figure 2D).

### Kaiso negatively regulates the expression of miR-31 by binding directly to its promoter

The expression and functional correlation between Kaiso and miR-31 led us to question if miR-31 is directly regulated by Kaiso. Thus we determined if the regulation of methylated miR-31 by Kaiso occurs in a transcriptionally dependent or independent manner. *In silico* analysis demonstrated that there are three possible Kaiso consensus binding sites and four methylation-dependent binding sites in the miR-31 promoter (Figure 3A). Seven primer sequences were designed for each of these sites (Supplementary Table 2) and the location of each is shown in (Figure 3B). ChIP assays were performed for all six putative Kaiso binding sequences (CKBS and MSBS) using ChIP-grade Kaiso 6F8 monoclonal antibody to investigate the association of Kaiso with the miR-31 promoter in PCa cells. Only the ChIP assay utilizing primer set 3 showed Kaiso binding to the upstream MSBS region of the miR-31 promoter, and this binding was abrogated in cells treated with 500 nM 5-aza-2′-deoxycytidine (5-aza) (Figure 3C). Kaiso binding to other methylation sites or KBS sites was not observed (Supplementary Figure 4).

### Inverse Kaiso/miR-31 expression suppresses PCa proliferation, migration and invasiveness

**Figure 4:**
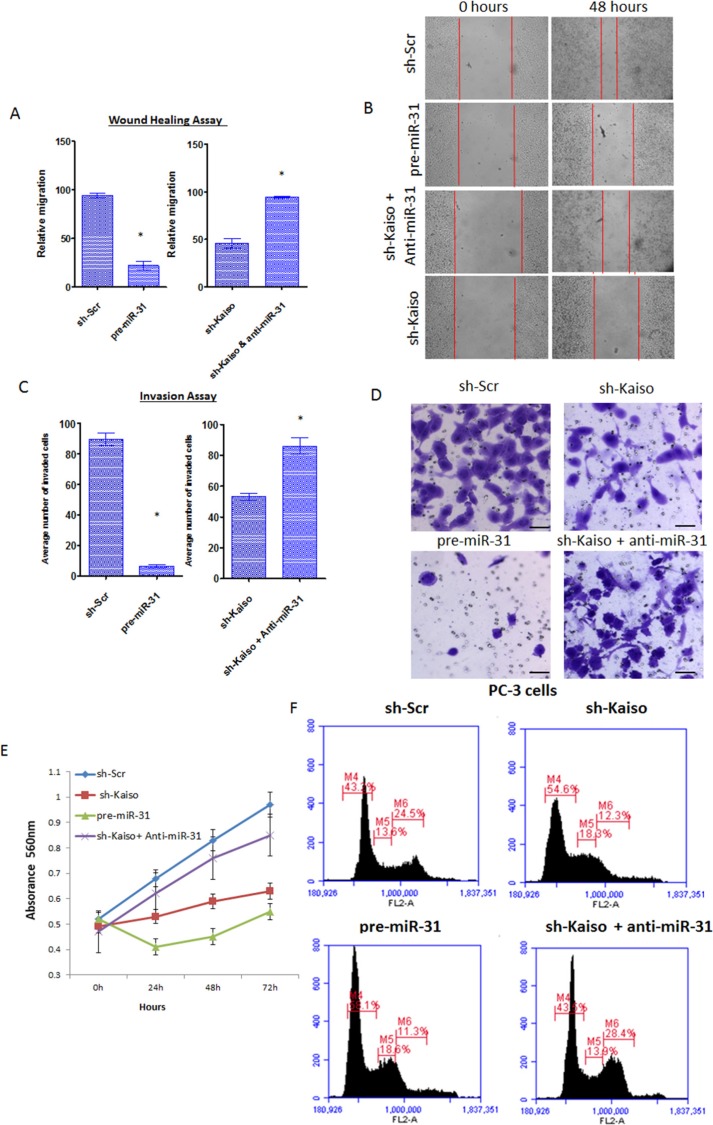
Kaiso regulates cell proliferation, migration and invasiveness, at least in part, through miR-31 (**A**) Quantitative analysis of PC-3 control cells or sh-kaiso transfected with pre-miR-31 or anti-miR-31 were assayed for cell migration relative to PC-3 sh-Scr control cells utilizing wound healing assays. (**B**) Photos were taken at X100 magnification. Red vertical bars indicate the starting area migration on day 0. All Bar graphs represent the average of three independent experiments, performed in triplicate ± S.E *p < 0.05. (**C**) Quantitative analysis of PC-3 control cells or sh-kaiso transfected with pre-miR-31 or anti-miR-31 were assayed for cell invasion relative to PC-3 sh-Scr control cells utilizing Matrigel invasion assay. Cells that penetrated the Matrigel coated membrane were fixed in formaldehyde and stained with crystal violet and counted. (**D**) Photos are representative fields of invasive cells on the membrane. Photos were taken at X100 magnification. All Bar graphs = 5 μM and represents the average number of cells on the underside of the membrane. All data presented are the means of three independent experiments, performed in triplicate ± S.E *p < 0.05. (**E**) Proliferation of PC-3 control cells or sh-kaiso transfected with pre-miR-31 or anti-miR-31 was measured by MTT for 4 days. Results shown are representative of three independent experiments averaged ± s.e. *p < 0.05. (**F**) PC-3 control cells or sh-kaiso transfected with pre-miR-31 or anti-miR-31 cells were analyzed by flow cytometry. Cell cycle phases are denoted M4 = G1, M5 = S, M6 = G2/M

To determine if the Kaiso/miR-31 relationship influences functional characteristics of aggressive PCa cells, i.e. cell migration and invasion, migration (wound healing) assays were performed. In contrast to PC-3 parental cells, PC-3 cells over-expressing miR-31 (Supplementary Figure 5) showed a decrease in migration into the wounded area 48 hrs after wounding (Figure 4A). Moreover, after an anti-miR-31 oligonucleotide was transfected into sh-Kaiso PC-3 cells, there was an increase in cell migration relative to sh-Scr PC-3 cells (Figure 4B). Similarly, assays were conducted in serum-containing medium to evaluate the capacity of PCa cells to invade through Matrigel. miR-31 over-expression inhibited migration of PC-3 control cells; however, sh-Kaiso PC-3 cells transfected with an anti-miR-31 oligonucleotide restored their invasive capacity (Figure 4C and 4D). To determine if sh-Kaiso or miR-31 has an influence on cell proliferation we similarly conducted MTT cell proliferation assay. We further measured cell proliferation in both our sh-Kaiso and miR-31 pretreated cells. As determined by MTT assay both pre-miR-31 and sh-Kaiso cells exhibited decreased cell proliferation compared to control. We further observed that sh-Kaiso cells treated with anti-miR-31 resumed proliferation similar to sh-Scr control cells (Figure 4E). Since sh-Kaiso and pre-miR-31 transfected cells demonstrated decreased proliferation, we next assayed these cells by flow cytometry to determine if there was an effect on cell cycle progression. sh-Kaiso treatment caused cells to accumulate at the G1 phase (sh-Kaiso 52.8.40% compared to sh-Scr cells 40.4%; pre-miR-31 treatment resulted in 56.6% compared to sh-Kaiso cells treated with anti-miR-31 42.0%; (Figure 4F). Thus, Kaiso/miR-31 expression influences the proliferative, migratory, and invasive capability of PCa cells.

### Kaiso and miR-31 are associated with decreased patient survival

**Figure 5:**
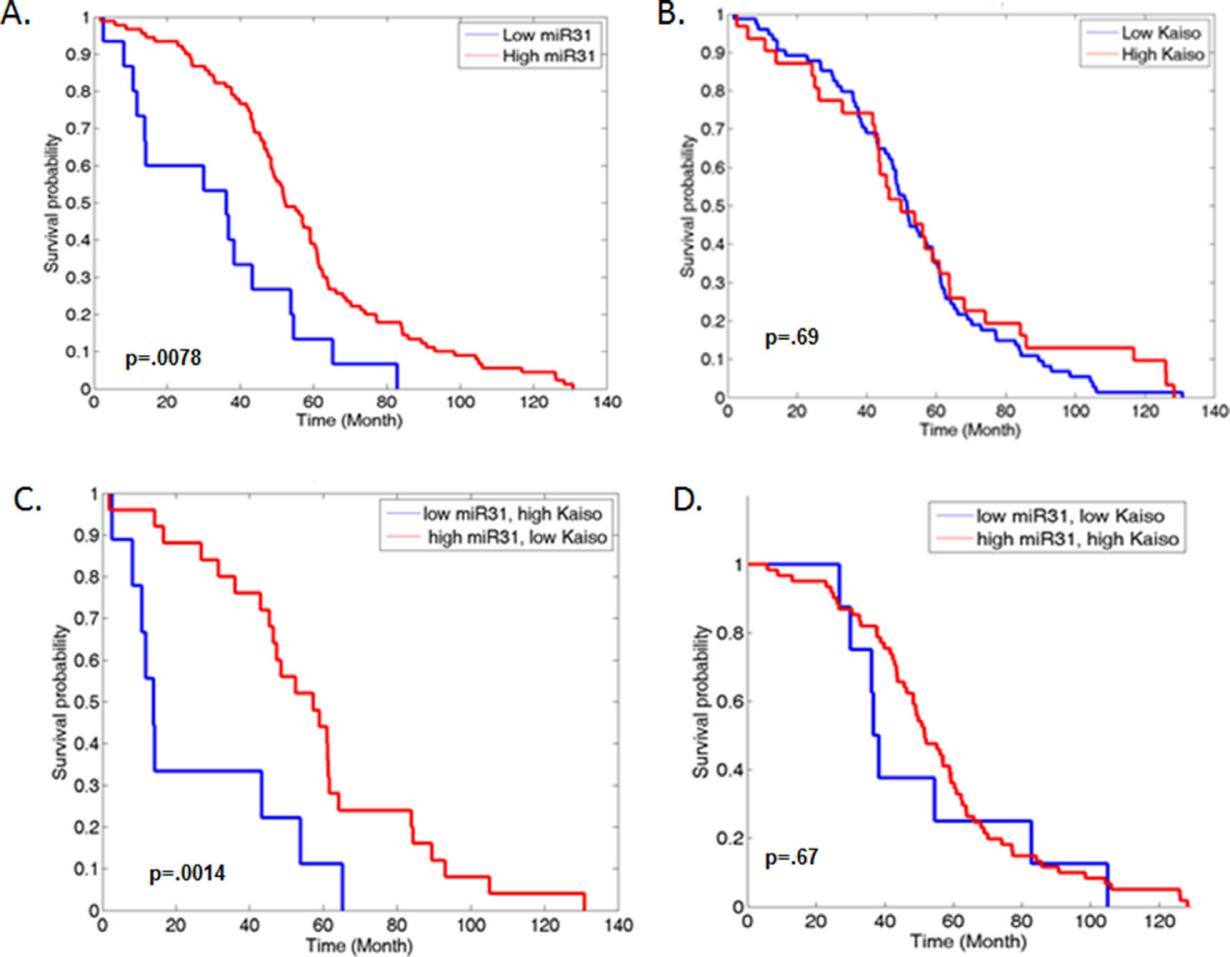
Increased expression of Kaiso and decreased miR-31 expression are correlated with decreased overall survival in PCa patients (**A**) Samples from the Taylor *et al.* (GEO accession number GSE21036) set were analyzed for patient survival, with low miR-31 compared to high miR-31 expression and plotted on a Kaplan-Meier curve (*p* = 0.0078). (**B**) Low Kaiso was compared to High Kaiso expression and plotted. (*p* = .69) (**C**) Low miR-31, high Kaiso compared to high miR-31, low Kaiso were plotted (*p* = 0.0014). (**D**) Low miR-31, low Kaiso compared to high miR-31, high Kaiso was plotted (*p* = .67).

We have demonstrated that Kaiso levels are increased in human PCa tissues [16, 21]. To determine the correlation of Kaiso expression and miR-31 in PCa, the effect of the Kaiso/miR-31 inverse expression was analyzed by use of the Taylor *et al.* GSE21032 data set available on the GEO website (http://www.ncbi.nlm.nih.gov/geo/). Patients with low miR-31 levels demonstrated reduced overall survival (*p* = 0.0078) (Figure 5A), compared to patient with high miR-31 levels. While patients with high levels of mRNA Kaiso levels did not show a significant difference compared to patient with low mRNA Kaiso levels (Figure 5B). We did observe that patients that had both high Kaiso levels and low miR-31 had the most significant decrease in survival (*p* = 0.0014) compared to patients with high miR-31, and low mRNA Kaiso levels (Figure 5C). We did not observe a significant difference in patients with low miR-31 and low mRNA Kaiso, or patient with high miR-31 or high mRNA Kaiso levels (Figure 5D). Thus, these results present a consistent picture that high Kaiso and low miR-31 levels are associated with poor PCa prognosis, and indicate that loss of miR-31 through Kaiso contributes to PCa progression.

## DISCUSSION

In various tumor types, promoter hypermethylation is responsible for decreased expression of various miRNAs, including miR-31 expression in PCa [26] and in triple-negative breast cancers [20]. We and others have demonstrated that Kaiso targets hypermethylated genes [10, 13, 16, 17, 21] and that silencing of Kaiso reverses the migration and invasiveness of metastatic PCa cell lines [16]. However, whether this holds true for methylated miRNA expression is an open question. Therefore, we utilized a two-miRNA microarray approach, with stringent cutoffs, to determine which of the most highly significant miRNAs where silenced by DNA methylation or by the presence of Kaiso in PC-3 cells. Our analysis revealed that 4 miRNAs (miR-9, miR-505, miR-636, and miR-31) were up-regulated after treatment with 5-aza or sh-Kaiso. Real-time PCR experiments validated that miR-31 is increased in stably transfected sh-Kaiso PC-3 cells, and ChIP analyses indicated that Kaiso binds to MSBS region covered by primer set 3 in the miR-31 promoter in a methylation dependent manner. Thus, these data provide evidence that Kaiso is involved in the direct regulation of miR-31 expression.

Aberrant expression of miR-31 can prevent metastasis by inhibiting the expression of multiple genes associated with various functions. For example, in breast cancer cells, miR-31 targets frizzled3 (*Fzd3*), integrin α-5 (*ITGA5*), myosin phosphatase-Rho-interacting protein (*M-RIP*), matrix metallopeptidase 16 (*MMP16*), radixin (*RDX*), and the ras homolog gene family member A (*RhoA*) [16, 22, 27]. Re-expression of *ITGA5* and *RDX* reverses the increased cell motility and invasiveness and anoikis resistance conferred by ectopic re-expression of miR-31 [22]. Interestingly, in our sh-Kaiso cells we observed significant decreases in MMP16 and Fzd3, however we observed the most significant decreases in SRC gene compared to sh-Scr control cells. In PCa, miR-31 also has a role in cell proliferation. miR-31 targets members of the E2F family of transcription factors (E2F1, E2F2, and E2F6) [26, 28, 29], and down-regulation of miR-31 results in elevated levels of E2F6, which in turn lead to resistance to chemotherapy-induced apoptosis [30].

Although miR-31 is decreased in prostate tumors relative to adjacent normal tissue, there are varying levels of decreased miR-31 expression in androgen receptor (AR)-positive PCa cells relative to AR-negative cells. In PCa cells, AR expression correlates with miR-31 promoter hypermethylation, and this results in a negative regulating loop [26]. In this report, we demonstrate that miR-31 is methylated in all PCa cell cancer lines tested, with C42B cells demonstrating more methylation relative to non-malignant RC-77N/E cells. Furthermore, this hypermethylation results in down-regulation of miR-31 expression in all malignant cells relative to normal PREC cells. Although LNCaP cells have higher promoter methylation relative to AR-negative DU-145 and PC-3 cells, all cell lines have sufficient methylation to result in down-regulation of miR-31 expression. Most importantly, increasing levels of Kaiso expression correlates with the loss of miR-31. DU-145 and PC-3 cells treated with sh-Kaiso have impaired migratory and invasive capabilities [16]. Kaiso-regulated miR-31 expression could be responsible for these observations, since inhibiting the re-expression of miR-31 in sh-Kaiso PC-3 cells restores the invasiveness of these cells. These findings are consistent with other studies, in which restoration of miR-31 levels in PC-3 and DU-145 cells inhibits cell invasion and migration [31]. Thus, Kaiso apparently has a mechanistic function in regulating miR-31 expression in PCa cells.

Other mechanisms have been proposed for the down-regulation of miR-31. EZH2, a methyltransferase involved in epigenetic silencing through H3K27 trimethylation (H3K27me3), is recruited to the miR-31 promoter region in LNCaP cells after stimulation with dihydrotestosterone. EZH2 serves as a recruitment platform for DNA methyltransferases, highlighting a previously unrecognized connection between two epigenetic repression systems [32]. However, it is unknown if this recruitment is specific to miR-31. DU-145 cells, which express Kaiso mainly in the cytoplasm, after transfection with Kaiso NLS mutant, fail to demonstrate decreased miR-31 expression relative to cells transfected with a functional Kaiso over-expression plasmid. Thus, it is possible that in these cells (which lack AR expression), growth factor signaling cascades such as EGFR are responsible for silencing of miR-31 through Kaiso localization. In support of this hypothesis are several lines of evidence. First, we found that among the most widely reported mRNA targets of miR-31, sh-Kaiso PC-3 cells demonstrated decreased levels of Src kinase expression. Similarly, in multiple melanoma cell lines which have lost miR-31 expression due to promoter hypermethylation, miR-31 pre-treatment results in decreased expression of Src and EZH2 [33]. In this report the authors further suggest that miR-31 activates cell signaling pathways that promote EZH2 expression and/or activity [33]. Second, we have reported that Kaiso expression and nuclear localization is influenced by stimulation of EGFR in both DU-145 and PC-3 cells [16]. Interestingly Src, which is activated by EGFR signaling, mediates cell proliferation of both DU-145 and PC-3 cells [34, 35]. While the mechanisms associated with activation of miR-31 and EZH2 are well studied in AR-positive cells [36–38], there are few studies of AR-negative PCa cells, which are in part driven by EGFR autocrine loops [39]. *EZH2* is 4–14-fold over-expressed in the androgen-refractory PC-3 and DU 145 cell lines, relative to the *EZH2* expression in the androgen-sensitive PZ-HPV-7 and LNCaP cells [40], suggesting that, in the absence of AR, Kaiso has a role in regulating EZH2 through miR-31 expression. PCa cells that lack AR apparently function as a source of tumor renewal, as these cells have stem cell characteristics and are resistant to hormonal therapy and chemotherapy [41–43]. More research needs to be conducted to understand the molecular mechanisms of epigenetic regulation of genes in this population of cells.

The miR-31/Kaiso regulatory mechanism has clinical implications. As determined with the Taylor *et al.* data set, low levels of miR-31 are associated with decreased survival. However, if patients with low miR-31 are further stratified with patients that have high Kaiso levels, there is an improvement in the ability to identify poor prognosis patients. A limitation of this study is direct comparison of miR-31 and Kaiso protein expression in individual patients and overall survival. However, since it is difficult to determine the expression of miRNAs correlated to protein expression in large patient cohorts, miRNA-mRNA expression patterns could be utilized to determine PCa patients with aggressive disease.

In summary, the miR-31-Kaiso regulatory mechanism could be responsible for the migratory and invasive ability of PCa cells. Furthermore, expression of the genes involved could be utilized as predictors of aggressive disease. Since Kaiso is over-expressed in PCAs and breast tumors of African Americans, miR-31-Kaiso correlative expression in tumors could be utilized as a marker to predict which tumors will progress, particularly tumors from African American patients, who have a higher incidence of aggressive disease.

## MATERIALS AND METHODS

### Cell culture, antibodies, and reagents

Human prostate cancer cell lines LNCaP, DU-145, C42-B and PC-3 were obtained from the ATCC and were cultured routinely in Dulbecco's modified Eagle's medium supplemented with 10% fetal bovine serum (Gibco, Paisley, Scotland) and antibiotics in a humidified chamber with 5% CO^2^. In these conditions the duplication period of the cells is 36 hours. Immortalized PCa cell lines RC-77T/E, RC-77N/E (non-malignant) were cultured in keratinocyte serum-free medium (KGM, LifeTechnologies, Carlsbad, CA) supplemented with bovine pituitary extract, recombinant epidermal growth factor, and 1% penicillin-streptomycin-neomycin as previously described [18, 19]. Non-malignant CA prostate epithelial cells (PrEC) were obtained from Clonetics Lonza (Switzerland) and maintained in Prostate Epithelial Cell Growth Medium (Clonetics). PREC, RC-77N/E, RC-77T, LNCaP and C42-B cells are positive for Androgen Receptor (AR). DU-145 and PC-3 cells are negative for Androgen receptor (AR).

### Profiling of miRNA expression

The Agilent Human miRNA Microarray Kit version 2 was used to profile miRNA expression. Total RNA (100 ng) was labeled using the Agilent miRNA Complete Labeling and Hybridization Kit (Agilent Technologies Incorporated, Santa Clara, CA, USA) according to the manufacturer's instructions. This array includes 723 mature human miRNAs based on the Sanger miRBase Release 10.1. Samples were hybridized onto an Agilent miRNA array, Human v2, at 55°C for 20 hr. After hybridization, slides were washed for 5 min in Gene Expression Wash Buffer 1 and then for 5 min in Gene Expression Wash Buffer 2, both at room temperature. Slides were scanned on an Agilent G2565A microarray scanner at 100% and 5% sensitivity settings. Agilent Feature Extraction software version 9.5.3 was used for image analysis. Arrays were scanned using an Agilent scanner and feature-extracted using Agilent Feature Extraction Software, version 10.5.1.1. TaqMan miRNA assays were used to quantify the levels of mature miRNAs following the manufacturer's protocol (Applied Biosystems).

### Quantitative real-time PCR (qRT-PCR)

Total RNA extraction was performed using the Ambion Recover All Nucleic Acid isolation kit (AM 1975) modified by replacing filters with ??(AM10066G). RNA (10 ng) was reverse transcribed using TaqMan miRNA reverse transcription kits (Life Technologies). For mRNA or miRNA expression, 1 μg of total RNA was reverse transcribed using High Capacity cDNA kits (Invitrogen). Relative expression of miRNAs and mRNA was quantified with the TaqMan Universal PCR Master Mix, No AmpErase UNG, with the 7500 Fast Real-Time PCR system (Life Technologies). Thermal cycling conditions included enzyme activation for 10 min at 95°C, 40 cycles of 95°C for 15 s, and 60°C for 60 s, according to provider's protocol. Reverse transcription for mRNA was accomplished as previously described [22, 38]. SYBR Green reagents (Invitrogen) were used for quantitative real-time PCR. Thermal cycling conditions for primer sequences used for mRNA detection are included in the table below. Analyses for miRNA and mRNA were performed in triplicate. RNU48 miRNA, GAPDH, and 18S ribosomal RNA were used as endogenous controls.

### Reagents for miR-31 transfection

Transient transfections of the miR-31 mature sequence, AGGCAAGAUGCUGGCAUAGCU; pre-miR-31 precursor (Ambion Product Number AM17100; Product ID: PM11465); or negative control miR (Ambion Product Number AM17110) were performed using Lipofectamine 2000 according to the manufacturer's protocol (Invitrogen). miR-31 expression was verified using TaqMan miRNA assays for qRT-PCR (mature miR-31 TaqMan microRNA Assay ID 002279).

### Chromatin immunoprecipitation assays

Chromatin immunoprecipitation (ChIP) experiments were performed with the use of ChIP-IT Kits (Active Motif, Carlsbad, CA). In brief, PC-3 cells were fixed in 1% formaldehyde at room temperature for 10 min; the fixation reaction was stopped by adding a 1:10 volume of 10 X glycine at room temperature for 5 min. The cell pellets were suspended and incubated for 30 min in ice-cold lysis buffer containing phenylmethylsulfonyl fluoride and a proteinase inhibitor cocktail. The nuclear pellets were suspended in shearing buffer, and chromatin was sheared to an average size of 200 to 800 bp by sonication at 25% power for 10 pulses of 20 sec, with a 30-sec rest on ice between each pulse. Chromatin (10 μL) was saved for input DNA control. Sheared chromatin was incubated in ChIP buffer 1 with 25 μL of protein G magnetic beads, anti-Kaiso antibody (Abcam), mouse RNA Polymerase II (Active Motif), and mouse IgG antibody (Active Motif, as a negative control) on a rolling shaker at 4°C for 4 hr. The immunoprecipitated chromatin was purified from the chromatin-antibody mixture by washing several times, and the ChIPed DNA was eluted in 50 μL of elution buffer AM2 (Active Motif). Crosslinks were reversed by adding 50 μL of reverse-crosslink buffer. After removing proteins by digestion with proteinase K, the purified DNA was used as a template for PCR analysis. The following primers specific for a region flanking the Kaiso binding sites were used for PCR: *consensus* Kaiso binding site (CKBS) primer sets 1, 6, and 7 and methylated CpG binding site (MSBS) 2, 3, 4, and 5 within the miR-31 promoter (Table S2).

### Bisulfite DNA modification and methylation-specific PCR

Genomic DNA (1–2 μg) was treated with bisulfite by use of (Zymo Research Corporation, CA) treatment kit. The treated DNA was recovered with the QIAquick Gel Extraction system (Qiagen, MA, USA), according to the manufacturer's instruction. The promoter-associated CpG island was predicted using the CpG prediction algorithm (http://www.urogene.org/methprimer/help.html#cpg_prediction) [20], and primers for methylation-specific PCR (Table S1) were designed by use of the Methprimer algorithm (http://www.urogene.org/methprimer/index.html).

### Cell proliferation

PC-3 cells (2.5 × 102) were plated in 96-well plates in DMEM. Cell viability was determined at 24, 48, and 72 hours. All cells were incubated with 10 μl of 3-(4,5-dimethylthiazol-2-yl)-2,5-diphenyltetrazolium bromide (MTT, Sigma-Aldrich, St. Louis, MO) solution (5 mg/ml) in PBS for 4 hr. Dimethylsulfoxide (50 μl) was added for 10 min after aspiration of the MTT solution. Plates were read at 560 nm.

### Cell migration

Migration of cells was assessed by their ability to move into an acellular area; this was accomplished with a two-dimensional wound-healing assay, as previously described [16, 21]. With cells at 70% to 80% confluence, a denuded area was generated in the middle of each well with a rubber policeman. The rate of migration was determined and quantified in Metamorph Imaging Software. All measurements were normalized to values for controls.

### Matrigel invasion assay

Cell invasiveness was determined by the capacity of cells to migrate across a layer of Matrigel in a Boyden chamber. Briefly, 20, 000 cells were plated in Matrigel-containing chambers in serum-free medium containing 1% bovine serum albumin for 24 hr; this medium was then replaced with serum-free medium for an additional 24 hr. The numbers of cells that invaded through the matrix over a 48-hr period were stained with crystal violet and determined by counting five random fields of the lower surface by use of an inverted microscope (Olympus IX51; Olympus America Inc., Melville, NY, USA). All experiments were performed in triplicate.

### Flow cytometry analysis

sh-Kaiso, pre-miR-31, and anti-miR-31 treated cells were harvested, washed with cold PBS, fixed, and permeabilized with 70% cold ethanol for propidium iodide staining. Cell cycle analysis was performed with a flow cytometer (Accuri, Ann Arbor, MI).

### Statistical analysis

For all experiments, statistics were performed with Prism software version 5.0 (GraphPad, La Jolla, CA). An independent Student's *t*-test was used to determine statistical differences between experimental and control values. The probabilities of overall survival were calculated using the Kaplan–Meier method and were compared using the log-rank test. For determining factors related to overall survival, a Cox proportional hazard model was utilized. Institutional Review Board of Tuskegee University approved the use of patient specimens for this study. Microarray arrays analysis was performed using SAS for Windows (Version 9.2; SAS Institute, Cary, NC). *P* values < 0.05 were considered statistically significant.

## SUPPLEMENTARY MATERIALS FIGURES AND TABLES


