# Supplementary material for: Kaiso, a transcriptional repressor, promotes cell migration and invasion of prostate cancer cells through regulation of miR-31 expression
Source: Oncotarget. 2015 Dec 30;7(5):5677–89. doi: 10.18632/oncotarget.6801 (PMC4868713; doi:10.18632/oncotarget.6801)
Supplement: Supplementary file 1 [file oncotarget-07-5677-s001.pdf]

Kaiso, a transcriptional repressor, promotes cell migration and invasion of prostate cancer cells through regulation of miR-31 expression

Supplementary Materials

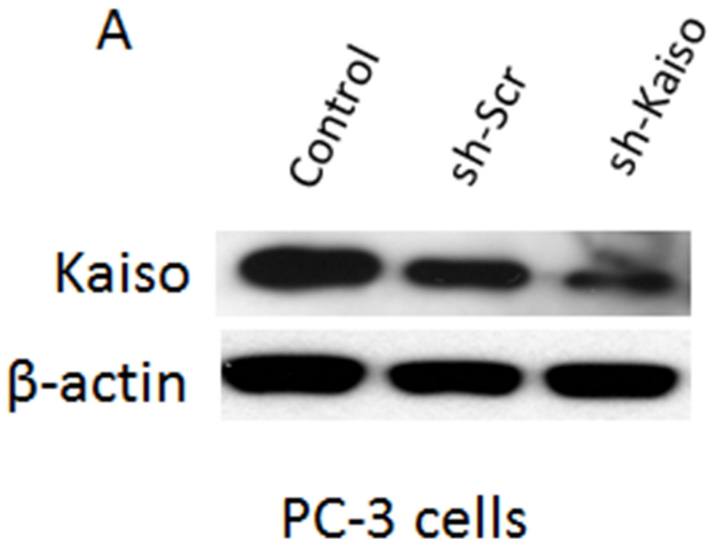

Supplementary Figure S1: PC-3 cells were transfected with sh-Kaiso plasmid and immunoblot was performed using Anti-Kaiso antibody. Beta-actin served as loading control.

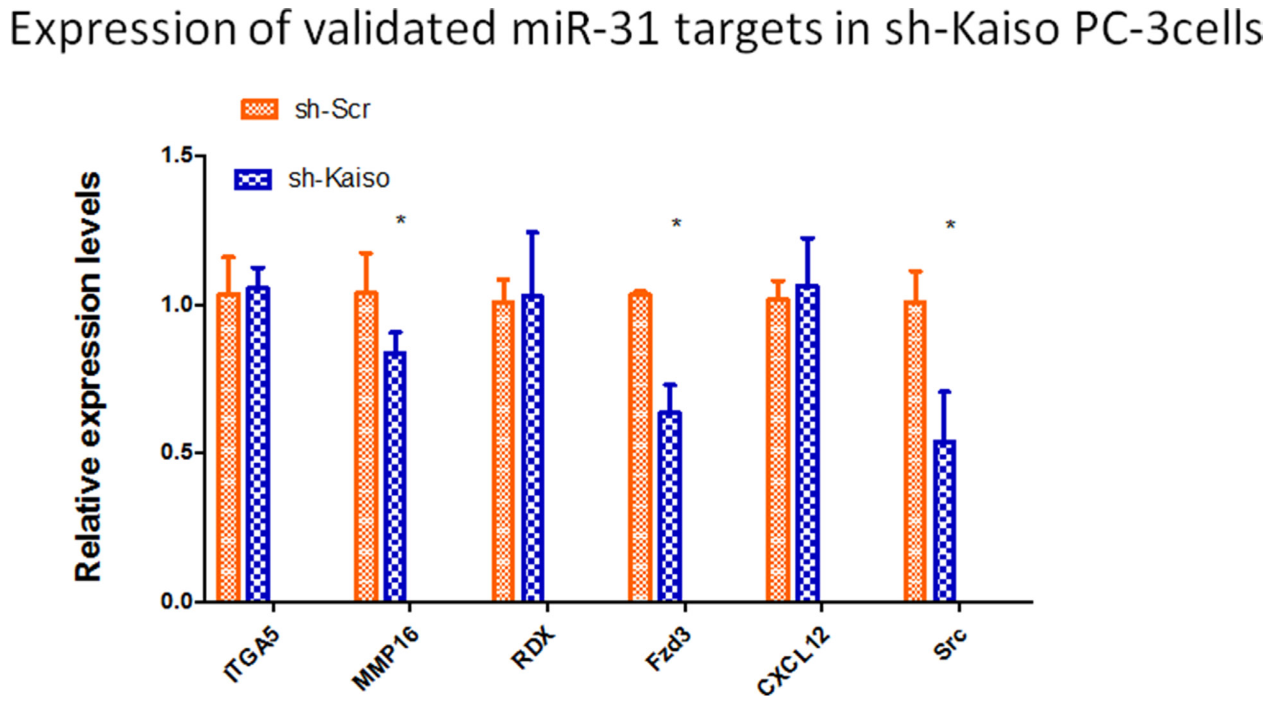

Supplementary Figure S2: qRT-PCR was performed on sh-Kaiso PC-3 cells compared to sh-Scr control cells for mRNA, ITGA5, MMP16, RDX, Fzd3, CXCL12, Src. Hypoxanthine-guanine phosphoribosyltransferase as used as the loading control.

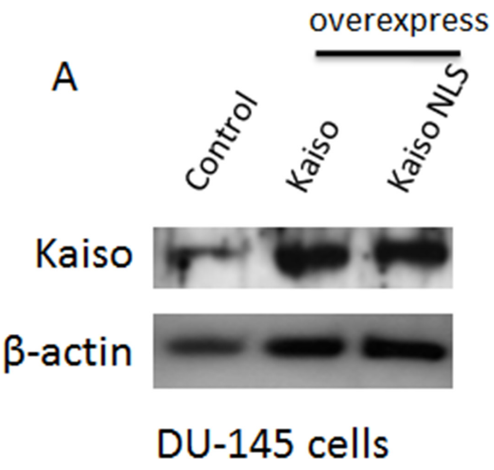

Supplementary Figure S3: DU-145 cells were transfected with Kaiso overexpression plasmid, Kaiso-NLS or the vector control. Immunoblot was performed using Anti-Kaiso antibody. Beta-actin served as loading control.

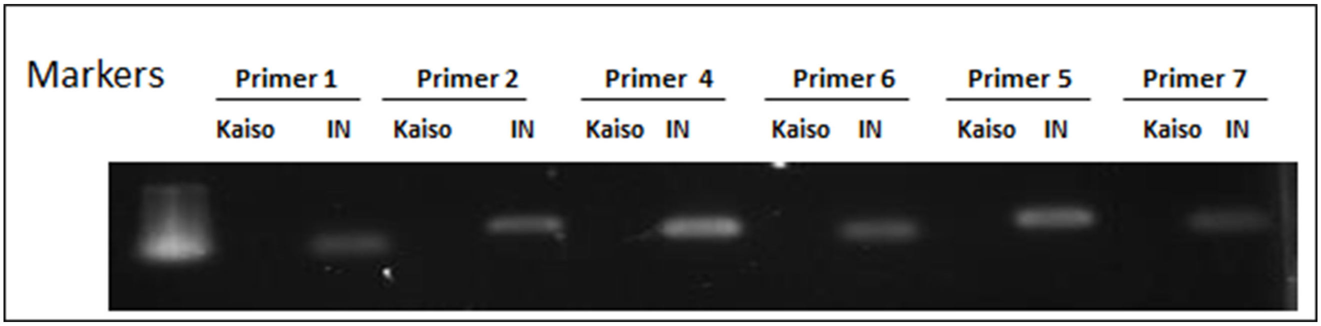

Supplementary Figure S4: Primer pairs 1, 2, 4, 5, 6, 7 that cover the miR-31 promoter region were assayed using ChIP analysis to determine the association between Kaiso protein and the miR-31 promoter. IN, input; Kaiso Ab, immunoprecipitation with Kaiso Ab.

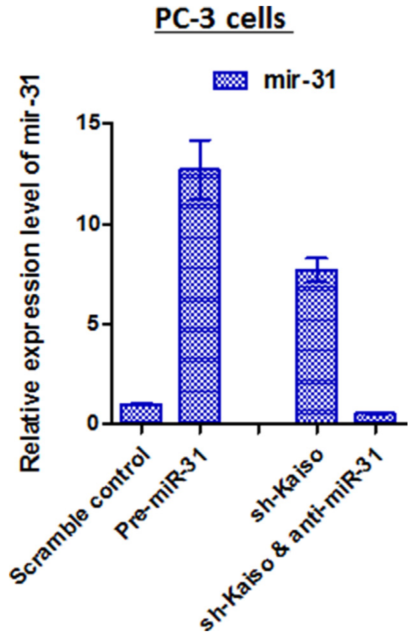

Supplementary Figure S5: miR-31 expression levels were determined in PC-3 control cells or PC3 sh-Kaiso cells transfected with pre-miR-31 or anti-miR-31 utilizing qRT-PCR with U6 as loading control.

Supplementary Table S1: Methylation specific PCR primers

31MSP-F1 TTTTAGTTAGTTTAGCGCGTAGC
31MSP-R1 CAAAACTAAAAAACGATATTCCGTA
31U-MSP-F1 TTTTAGTTAGTTTAGTGTGTAGTGG
31U-MSP-R1 CAAAACTAAAAAACAATATTCCATA
31MSP-F2 TTCGGTTTCGTTTAAGCGTATAC
31MSP-R2 AAAACTCGCAAATTCCACGT
31U-MSP-F2 TTTGGTTTTGTTTAAGTGTATATGA
31U-MSP-R2 CAAAAACTCACAAATTCCACATC
31MSP-F3 TCGATTGTCGTGTGTTTATTGTC
31MSP-R3 AAACGACCCCTTCGTATACG
31U-MSP-F3 TTGATTGTTGTGTGTTTATTGTTGT
31U-MSP-R3 AAAAACAACCCCTTCATATACACT
31MSP-F4 CGGGATTTAGGTTTTTTTATTGTAAC
31MSP-R4 CCTCTCCCTTAACTCTAACTACGAA
31U-MSP-F4 TGGGATTTAGGTTTTTTTATTGTAATG
31U-MSP-R4 CCTCTCCCTTAACTCTAACTACAAA

Supplementary Table S2: Primers specific for a region flanking the Kaiso binding sites (KBS—primer set 1, 6, 7 and MSBS 2,3,4, 5)

MiR31CHIPF1	AAGGGGTCACTTTTGCCTAA
MIR31CHIPR1	TTGCCCTGTACAGCTGAGAA
MiR31CHIPF2	TTCCTCGAGGTTTAGGGACA
MIR31CHIPR2	AAGGAGTTGAGGAGGCTTCG
MIR31CHIPF3	GAAGCCTCCTCAACTCCTTG
MIR31CHIPR3	GCGCTTCTGTCCTCCTACTC
MiR31CHIPF4	CCGAGTAGGAGGACAGAAGC
MIR31CHIPR4	TGCGACCTGTGCATAACTTG
MiR31CHIPF5	CCCCAAGTTAT G CACAG GTC
MIR31CHIPR5	AAGGGAGGAGGCTCAGCAC
MiR31CHIPF6	AAAGGAGGGGGAGGGAAG
MIR31CHIPR6	GCTCTGACTGCGAGAAGCTC
MiR31CHIPF7	TTTTGGCACAGTAGCCCTTT
MIR31CHIPR7	GAGAGAGGCCACTCCAGATG
